# How much of skin improvement over time in systemic sclerosis is due to normal ageing? A prospective study with shear-wave elastography

**DOI:** 10.1186/s13075-020-02150-x

**Published:** 2020-03-18

**Authors:** T. Santiago, M. Santiago, M. Coutinho, M. J. Salvador, J. A. P. Da Silva

**Affiliations:** 1 Rheumatology Department, Centro Hospitalar e Universitário de Coimbra, Coimbra, Portugal; 2 Faculty of Medicine, University of Coimbra, Coimbra, Portugal; 3 Coimbra Institute for Clinical and Biomedical Research (iCBR), Faculty of Medicine, University of Coimbra, Coimbra, Portugal

**Keywords:** Skin, Stiffness, Shear-wave elastography, Systemic sclerosis, Ultrasound

## Abstract

**Background:**

Measurement of skin involvement is essential for the diagnosis and assessment of prognosis and disease progression in systemic sclerosis (SSc). The modified Rodnan skin score (mRSS) is the gold standard measure of skin thickness, but it has been criticised for the lack of objectivity, poor inter-observer reproducibility and lack of sensitivity to change. Recently, shear-wave elastography (SWE) emerged as a promising tool for the objective and quantitative assessment of the skin in SSc patients. However, no studies have evaluated its sensitivity to change over time.

**Objective:**

To assess changes in skin stiffness in SSc patients using SWE during a 5-year follow-up.

**Methods:**

Skin stiffness [i.e. shear-wave velocity values (SWV) in metres per second] was assessed by SWE ultrasound (using virtual touch image quantification) at the 17 sites of the mRSS, in each participant, at baseline and follow-up. mRSS was performed at both time points. Differences between groups were analysed using the related-samples Wilcoxon signed-rank test and the Mann–Whitney *U* test.

**Results:**

We included 21 patients [85.7% females; mean age 56.3 (10.4) years at baseline, 57.1% with limited SSc] and 15 healthy controls [73.3% females; mean age 53.6 (14.1) years)]. The median follow-up was 4.9 (0.4) years.

Skin stiffness decreased significantly at all Rodnan sites (*p* ≤ 0.001) (except in the fingers), in SSc patients, over time. The same phenomenon occurred in controls, but to a lesser degree, in terms of percentage change.

The percentage reduction in skin stiffness varied in the different Rodnan sites and in different phases of the disease. In addition, SWV values also decreased significantly in 15/16 skin sites with local normal Rodnan at baseline, whereas local Rodnan skin score only changed significantly in the upper arm (*p* = 0.046) and forearm (*p* = 0.026).

**Conclusion:**

This study provides first-time evidence suggesting that skin SWV values are more sensitive to change over time than mRSS and reduce significantly over time in SSc and normal controls.

## Introduction

Skin involvement is a major feature of systemic sclerosis (SSc) [1]. The extent and rate of progression of skin fibrosis is of paramount importance as it correlates with functional limitations, internal organ involvement and survival [1]. Therefore, measurement of skin involvement is not only essential for the diagnosis and assessment of prognosis in SSc, but also crucial to support the development of new therapies. The modified Rodnan skin score (mRSS), a semi-quantitative method based on palpation, is currently the gold standard measure of skin changes in SSc and is often the primary or secondary outcome measure in clinical trials. However, it has been criticised for its lack of objectivity, poor inter-observer reproducibility and lack of sensitivity to change in skin thickness over time [2, 3].

Different ultrasound methods are being investigated as means to improve the assessment of skin involvement in SSc. High-frequency ultrasound offers a potential for objective, sensitive and reliable assessment of *dermal thickness* in SSc [4–6]. However, it does not assess the tissue elastic properties.

In recent years, shear-wave elastography (SWE) has been investigated as a quantitative and operator-independent tool to evaluate skin stiffness [7–9]. Shear-wave velocity (SWV) values reflect tissue stiffness: the stiffer the tissue, the faster the shear-waves propagate. SWE may, therefore, provide a novel opportunity to objectively assess fibrosis—a crucial feature in the complex process of skin involvement in SSc [10, 11].

Cross-sectional studies have shown that SWV values are significantly higher in SSc patients than in controls, in almost all of the Rodnan sites [7–9]. Interestingly, clinically unaffected skin of patients with SSc could also be differentiated from the skin of healthy comparators [7, 8]. Two previous studies have shown excellent reproducibility for SWV measurements, with inter-rater intraclass correlation coefficients (ICCs) ranging from 0.48 (phalanx) to 0.91 (upper arm). The corresponding values for intra-rater comparisons were 0.48 (chest) to 0.98 (phalanx) [7, 8].

This is the first study to evaluate the progression of skin stiffness over time with SWE in patients with SSc and in normal controls.

## Methods

### Participants

All participants were recruited from a cross-sectional evaluation previously described elsewhere [9]. In this longitudinal study, we included 21 of the original 26 patients (3 died and 2 were lost to follow-up) and 15 of the 17 initial healthy controls (1 died and 1 was lost to follow-up). The healthy controls were recruited among hospital staff and patient’s family members, using as exclusion criteria, any diagnosis of other skin disorders (e.g. psoriasis), connective tissue disease or rheumatic inflammatory disease. No significant differences were found between patients and controls, regarding age and gender.

All participants were submitted to a clinical and ultrasound evaluation at baseline and at follow-up, a median of 4.9 (0.4) years later.

All SSc patients fulfilled the 2013 ACR/EULAR criteria for the classification of SSc [12]. The disease was classified as diffuse cutaneous or limited cutaneous SSc, according to the extent of skin involvement [13].

#### Ethics

Ethical approval was obtained from the Ethics Committee of Centro Hospitalar e Universitário de Coimbra (CHUC – 118-17). All patients and controls provided signed informed consent prior to any study procedures.

#### Clinical skin thickness scoring (mRSS)

Skin thickness was clinically assessed using the mRSS, scoring the palpation at each of the 17 skin sites on a 0–3 scale [14]. The same experienced rheumatologist (MJS) performed the mRSS at baseline and follow-up, on the same day of the skin ultrasound.

#### Clinical phase of skin involvement

Skin involvement phase was clinically assessed and classified as oedematous, fibrotic or atrophic by the same rheumatologist (MJS) at baseline and follow-up, on the same day of the skin ultrasound, following currently accepted descriptions [15–18].

#### Skin ultrasound evaluation

Skin stiffness was measured at baseline and follow-up, through shear-wave elastography, using virtual touch image quantification (VTIQ), at the same 17 sites of the mRSS. SWE was performed with an ACUSON Ultrasound System (Siemens Healthcare), using a linear 4–9-MHz transducer. The ultrasound protocol has been described elsewhere [7]. In brief, acceptance of an ultrasound image for analysis was based on clear visualisation of an interface between the epidermis, dermis and subcutaneous tissues and on an automated image quality indicator provided by the ultrasound system. The sonographer placed sampling gates with the minimum possible size (2 × 2 mm) over the dermis. The VTIQ output simultaneously displays a colour-coded tissue stiffness map and absolute shear-wave velocity values (in metres per second, up to 10 m/s) in one single image. Higher shear-wave velocities indicate greater tissue stiffness. The SWV for each site scanned was established as the mean of three consecutive measurements.

The same rheumatologist (TS) performed all ultrasound measurements, blinded for the attributed Rodnan skin score. The intra-observer reproducibility of SWE in this examiner’s hands is reflected by intraclass correlation coefficients ranging from 0.70 (foot) to 0.98 (finger) in SSc and 0.81 (thigh) to 0.97 (finger) in healthy controls (Table S1).

#### Statistical methods

Continuous variables were reported as means (standard deviation), if normally distributed or median (interquartile range), if not normally distributed. Categorical variables were presented as frequencies. Differences between groups were analysed using the related-samples Wilcoxon signed-rank test and the Mann–Whitney *U* test.

## Results

### Clinical features

Baseline clinical features of the patients with SSc and healthy controls are presented in Table 1. All patients in an oedematous phase at baseline progressed to a fibrotic (*n* = 3) or atrophic phase (*n* = 2). Of the 16 patients in a fibrotic phase at baseline, 11 maintained the fibrotic phase and 5 progressed to an atrophic phase.

**Table 1:** Clinical and demographic characteristics of the participants at baseline

	SSc patients (*N* = 21)	Controls (*N* = 15)
**Female,*n*(%)**	18 (85.7)	11 (73.3)
**Age (years)**	58.0 (48.5–63.0)	55.0 (45.0–63.0)
**Smoking habits,*n*(%)**		
**Never**	17 (80.9)	11 (73.3)
**Ex-smoker**	4 (19.1)	4 (26.6)
**Disease duration since diagnosis, years**	10.0 (5.5–14.0)	–
**Disease duration since RP, years**	14.0 (6.5–16.5)	–
**Limited form,*n*(%)**	12 (57.1)	–
**mRSS total**	8.0 (4.0–15.0)	–
**Phase,*n*(%)**		–
**Oedematous**	5 (23.8)	
**Fibrotic**	16 (76.2)	
**ANA positive,*n*(%)**	20 (95.2)	–
**ACA positive,*n*(%)**	9 (42.9)	–
**Anti-Scl 70 positive,*n*(%)**	7 (33.3)	–
**Immunosuppressive treatment**^a^**(yes),*n*(%)**	6/21	–
**Vasodilators treatment**^**b**^**(yes),*n*(%)**	9/21	–

Values are in median (Q1–Q3), unless stated otherwise

*RP* Raynaud phenomenon, *ANA* anti-nuclear antibody, *ACA* anti-centromere antibody, *mRSS* modified Rodnan skin score

^a^Methotrexate (average dose 15 mg/week, *N* = 2); prednisolone or equivalent (average dose 5 mg/day (*N* = 4)

^b^Nifedipine (average dose 30 mg/day, *N* = 7) and/or pentoxifylline (average dose 800 mg/day, *N* = 5)

### Changes in skin stiffness during follow-up

Significant decreases in SWV values were observed in all Rodnan skin sites over the follow-up period (*p* ≤ 0.001), except in the fingers (Table S2). mRSS only identified significant changes in the upper arm (*p* = 0.046) and forearm (*p* = 0.024) (Table S2).

Similar significant decreases in SWV values were observed in healthy controls in all skin sites (*p* = 0.001), except the leg.

At the second examination, SWVs in SSc patients became similar to that of controls in all sites, except the hands and fingers (*p* = 0.001) (Table S2).

**Fig. 1:**
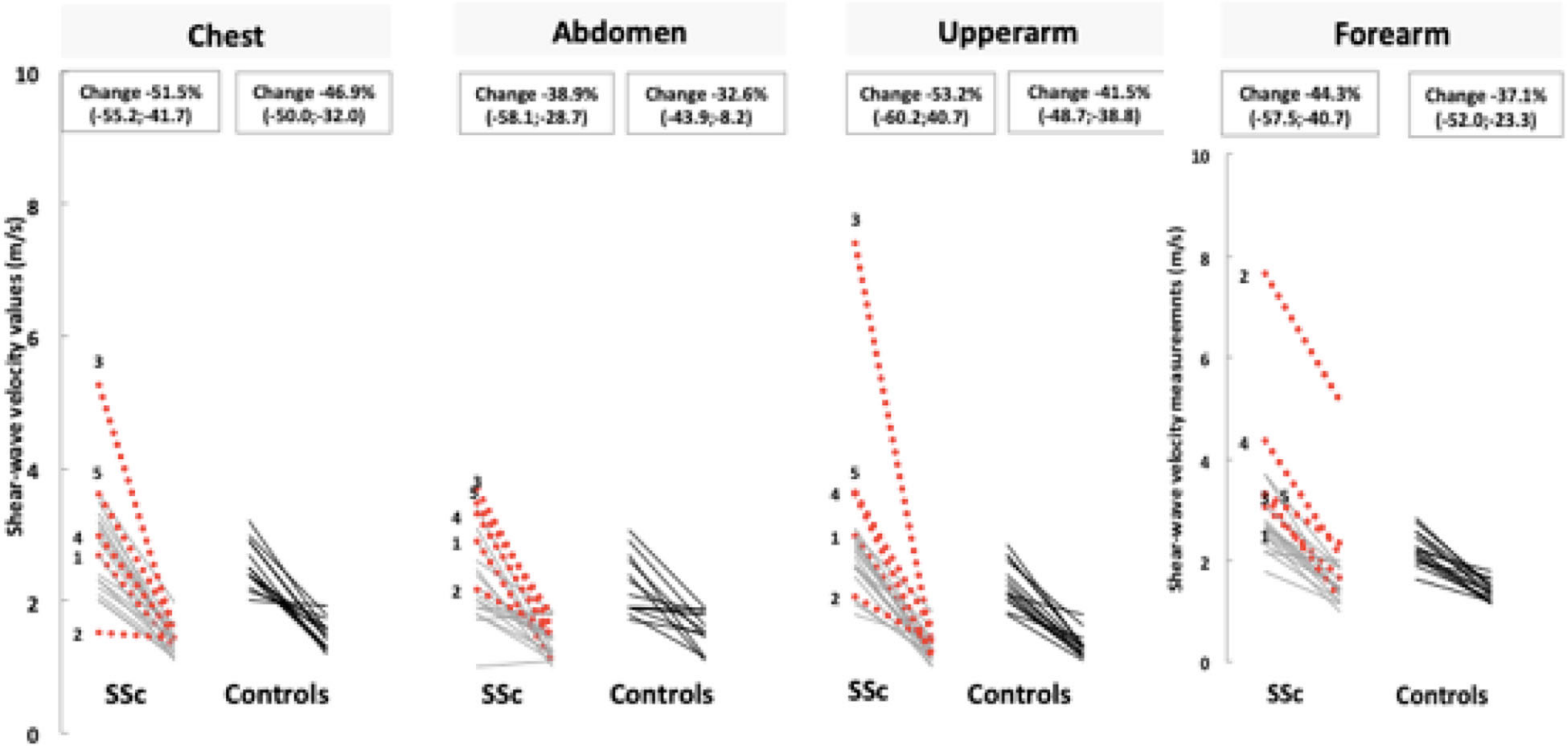
Shear-wave velocity values (metres per second), measured by shear-wave elastography, at the Rodnan skin sites, at baseline and follow-up, in SSc and controls. Percentage change values are presented as median (Q1–Q3). Patients 2 and 3 progressed from oedematous to atrophic phase. Red dotted lines represent oedematous patients at baseline, and the grey lines represent patients in fibrotic phase at baseline

The median percentage change in skin stiffness (i.e. percentage change of SWV from baseline) was more pronounced in SSc than in controls. This difference reached statistical significance in the upper arm (median percentage change − 53.2% in SSc vs − 41.5% in controls, *p* = 0.007) (Fig. 1 and Table 2). In addition, the percentage change of SWV was variable in different skin sites (Table 2).

**Table 2:** Comparison of percentage changes in shear-wave velocity values, in each Rodnan site of analysis, observed in SSc patients and controls

Rodnan sites	SSc patients (*n* = 21)^#^	Controls (*n* = 15)^#^	SSc vs controls (*p* value)^**†**^
**Chest**	− 51.5% (− 55.2 to − 41.7)	− 46.9% (− 50.0 to − 32.0)	NS
**Abdomen**	− 38.9% (− 58.1 to − 28.7)	− 32.6% (− 43.9 to − 8.2)	NS
**Upper arm**	**− 53.2% (− 60.2 to − 40.7)**	**− 41.5% (− 48.7 to − 38.8)**	**0.007**
**Forearm**	− 44.3% (− 57.5 to − 40.7)	− 37.1% (− 52.0 to − 23.3)	NS
**Hand**	− 33.5% (− 52.6 to − 19.9)	− 26.2% (− 38.6 to − 8.5)	NS
**Finger**	− 5.3% (− 36.9 to 34.7)	− 24.6% (− 29.8 to − 12.4)	NS
**Thigh**	− 37.1% (− 45.3 to − 31.2)	− 31.6% (− 37.3 to − 24.0)	NS
**Leg**	− 25.8% (− 37.8 to − 3.2)	− 10.3% (− 27.3 to − 6.9)	NS
**Foot**	− 36.4% (− 56.6 to − 29.7)	− 27.1% (− 45.2 to − 14.3)	NS

Statistically significant results are in bold

*NS* non-significant

^#^Values are in medians (Q1–Q3)

^†^Mann–Whitney *U* test

Taking all cases into account, and based in simple arithmetic, the effects of ageing seem to explain from 40% (leg) to 90% (chest) of the skin stiffness reduction observed in SSc patients. The only exception is the skin of the fingers, where the disease is associated with a smaller decrease in skin stiffness than observed in healthy controls (data not shown).

### Skin stiffness and its progression according to the clinical phase of the disease

Patients in an oedematous phase had higher SWV compared to patients in a fibrotic phase. These differences were statistically significant at the abdomen, upper arm, forearm, hand and foot (*p* < 0.05).

The percentage change differed according to the phase of the disease at baseline (Table S3 and Supplementary Fig. S1). Namely, patients in an oedematous phase at baseline had a higher percentage reduction in skin stiffness, in the majority of skin Rodnan sites, than patients in a fibrotic phase.

#### Changes in skin stiffness according to the form of the disease

At baseline and follow-up, patients with a diffuse form had higher SWV values than patients with a limited form (Table S6). These differences were statistically significant at the upper arm, hand and finger (*p* < 0.05). However, there were no statistically significant differences in percentage change reduction in skin stiffness between patients with the limited and diffuse forms of SSc.

### Changes in skin stiffness in sites with normal mRSS

The observation of higher SWV values compared with controls in sites with clinically unaffected skin (mRSS = 0) at baseline made in our original study was confirmed in this subgroup [7] (Table S4 and Table S5).

The longitudinal analyses demonstrated that SWV values also decreased significantly over the 5 years follow-up in all skin sites with Rodnan = 0 at baseline (except in the fingers). There were no statistically significant differences between patients and controls at the end of follow-up in any of these sites (Table S4). Naturally, the Rodnan skin score could not identify any changes in such sites.

## Discussion

This is the first study evaluating changes of skin stiffness over time in patients with SSc, using shear-wave elastography. This study provides evidence suggesting that skin stiffness (i.e. SWV values) decreased significantly in almost all Rodnan skin sites in SSc patients, as well as in healthy controls, over 5 years of follow-up. Shear-wave elastography was remarkably more sensitive to change over time than mRSS.

The observed decrease in stiffness follows the classical clinical expectation that skin in SSc evolves from an early oedematous status towards a fibrotic and finally an atrophic phase after reaching a maximal induration [15, 16]. In fact, at baseline, the five patients in the oedematous phase had higher SWV values than patients in a fibrotic phase in the corresponding skin sites. During follow-up, SWV values decreased in almost all skin sites, which parallels the decline of oedema, the onset of fibrosis and, finally, atrophy.

Surprisingly, however, our observations in healthy controls suggest that a substantial part of the decrease in skin stiffness observed in patients with SSc is probably explained by normal skin ageing. Collagen fibre network of the dermis layer is known to change with ageing and this is expected to affect the elasticity of this layer [18]. In fact, Shuster et al. measured the skin collagen and dermal thickness in skin biopsies obtained from the forearm of ~ 150 healthy controls [18]. They demonstrated that skin collagen decreased with age, namely after the age of 20 in males and 50 in females [18]. Another study by Leveque et al. found that skin thickness starts to decrease from the age of 45 years both in male and female, with the female’s skin becoming thinner than that of males [19]. Interestingly, these findings were recently corroborated by a study using SWE to determine age-related changes of the skin in healthy controls [20]. These authors demonstrated that SWV values decrease significantly in healthy controls older than 50 years compared with the 20- to 50-year group, at the finger and forearm [20]. As the previously mentioned studies were cross-sectional, we cannot infer skin changes over time from them [18–20]. Of note, in the present study, 72.2% of the participants were older than 50 at baseline [60.3 (7.7) years]. Other factors, besides age itself, such as skin site, gender, hormonal phase and contextual factors, may have also contributed to the observed changes and deserve consideration in future studies.

Our results suggest that elastography may be useful as an aid in distinguishing between changes in the skin due to oedema and induration or sclerosis, a recognised limitation of mRSS [15]. This may be particularly important in the assessment of the early phases of disease and response to treatment. Similar observations have been made in two longitudinal studies of ultrasound dermal thickness: thickness decreased and patients became more similar to the control population, between the 1st and the 4th years of follow-up [4, 21]. Kaloudi et al. found that dermal thickness decreased as the clinical phase progressed from the oedematous to the atrophic phase [6].

A relevant key message from our findings provides the evidence that skin SWV evaluation is a more sensitive instrument to measure skin change over time than mRSS. In fact, SWE identified significant changes over time at all skin sites (except fingers), where mRSS only showed significative differences in the upper arm and forearm.

Another key message is the fact that SWE captured significant changes over time in skin sites with local normal mRSS at baseline. This is reinforced by the obvious fact that mRSS would, by definition, be unable to identify age-related skin changes in normal skin and, thus, the impact of ageing in SSc.

These comparisons should, however, be interpreted in light of evidence that the mRSS and SWE measure different skin properties: mRSS measures not only thickness, but also texture and fixation [15], while elastography measures only skin stiffness. In future studies, it would be of interest not only to validate SWE against dermal thickness ultrasound or optical coherence tomography, but also against histologic findings.

We also observed that percentage SWV reduction was more pronounced in certain sites (chest, upper arm, and forearms) than in others. This is in line with studies that have identified the chest and forearms as the sites with more pronounced skin changes over time, as opposed to the lower extremities, abdomen, fingers, and face, which tend to be more stable [22]. These findings raise the hypothesis that excluding relatively static skin sites may improve the sensitivity to change of total skin scores.

This is the first study addressing the sensitivity over time of SWE in SSc and controls. The same observers performed ultrasound evaluations and mRSS at baseline and follow-up. Although our data further supports the use of SWE as a potential outcome measure of skin involvement in SSc, its interpretation is limited by the small sample size, forcing a more descriptive than statistical subgroup analysis. Future skin ultrasound studies would benefit from a cohort of early diffuse patients with a shorter evaluation interval to further clarify whether changes are age- or disease-related, compared to later disease and healthy controls. It should also be considered that about half of the patients received immunosuppressive treatment between the two clinical and ultrasound evaluations: it cannot be ruled out that some of the changes observed were influenced by these medications.

## Conclusions

In conclusion, findings reported herein highlight that a substantial part of the improvement of the skin in SSc may be explained by normal ageing. They support the higher discriminant ability of shear-wave elastography in detecting subtle skin changes not identified by mRSS. Further longitudinal studies with a higher number of patients in different phases of skin involvement are needed to fully clarify its potential. Establishing normal reference data for these ultrasound measurements may also foster earlier diagnosis.

## Supplementary information

**Additional file 1: Table S1.** ICCs of intra-observer reproducibility of skin stiffness ultrasound measurements performed by the US examiner responsible for this study. **Table S2.** Shear-wave velocity values and mRSS, per site of analysis, at baseline and follow-up, in SSc patients and controls. **Table S3.** Shear-wave velocity values, at Rodnan skin sites, between baseline and follow-up, in the patients with SSc according to clinical skin phase of the disease. **Table S4**. Shear-wave velocity values at Rodnan sites with clinically unaffected skin (i.e. mRSS=0) and in controls, at baseline and follow-up. **Table S5**. Percentage changes in shear-wave velocity values at Rodnan sites with unaffected skin at baseline (i.e. mRSS=0) and in controls, between baseline and end of follow-up. **Table S6**. Shear-wave velocity values, at Rodnan skin sites, at baseline and follow-up, in patients with limited vs diffuse SSc. **Figure S1.** Shear-wave velocity values in the skin Rodnan sites at baseline and follow-up, in SSc patients, according the progression of the skin phase of the disease. Patients in an oedematous phase progressed to a fibrotic (*n*=3) or atrophic phase (*n*=2); 11 maintained the fibrotic phase; and 5 progressed from a fibrotic to an atrophic phase. Percentage change values are in medians (Q1-Q3).

## Data Availability

All data relevant to the study are included in the article or uploaded as supplementary information.

## Abbreviations

CHUC: Centro Hospitalar e Universitário de Coimbra
mRSS: Modified Rodnan skin score
SSc: Systemic sclerosis
SWE: Shear-wave elastography
SWV: Shear-wave velocity
VTIQ: Virtual touch imaging and quantification
