# Supplementary material for: How much of skin improvement over time in systemic sclerosis is due to normal ageing? A prospective study with shear-wave elastography
Source: Arthritis Res Ther. 2020 Mar 18;22:50. doi: 10.1186/s13075-020-02150-x (PMC7079468; doi:10.1186/s13075-020-02150-x)
Supplement: Supplementary file 1 — Additional file 1: Table S1. ICCs of intra-observer reproducibility of skin stiffness ultrasound measurements performed by the US examiner responsible for this study. Table S2. Shear-wave velocity values and mRSS, per site of analysis, at baseline and follow-up, in SSc patients and controls. Table S3. Shear-wave velocity values, at Rodnan skin sites, between baseline and follow-up, in the patients with SSc according to clinical skin phase of the disease. Table S4. Shear-wave velocity values at Rodnan sites with clinically unaffected skin (i.e. mRSS=0) and in controls, at baseline and follow-up. Table S5. Percentage changes in shear-wave velocity values at Rodnan sites with unaffected skin at baseline (i.e. mRSS=0) and in controls, between baseline and end of follow-up. Table S6. Shear-wave velocity values, at Rodnan skin sites, at baseline and follow-up, in patients with limited vs diffuse SSc. Figure S1. Shear-wave velocity values in the skin Rodnan sites at baseline and follow-up, in SSc patients, according the progression of the skin phase of the disease. Patients in an oedematous phase progressed to a fibrotic (n=3) or atrophic phase (n=2); 11 maintained the fibrotic phase; and 5 progressed from a fibrotic to an atrophic phase. Percentage change values are in medians (Q1-Q3). [file 13075_2020_2150_MOESM1_ESM.doc]

**SUPPLEMENTARY TABLES AND FIGURE 2**

**Table S1-** ICCs of intra-observer reproducibility of skin stiffness ultrasound measurements performed by the US examiner responsible for this study.

| Skin sites | **SSc** | **Controls** |
| --- | --- | --- |
| **Face** | 0.89 (0.74 – 0.96) | 0.92 (0.79-0.97) |
| **Chest** | 0.92 (0.79 – 0.97) | 0.92 (0.82-0.97) |
| **Abdomen** | 0.90 (0.74-0.96) | 0.88 (0.66-0.95) |
| **Upperarm** | 0.90 (0.73-0.96) | 0.88 (0.65-0.96) |
| **Forearm** | 0.97 (0.94-0.99) | 0.94 (0.80-0.97) |
| **Hand** | 0.90 (0.75-0.96) | 0.83 (0.50-0.93) |
| **Finger** | 0.98 (0.96-0.99) | 0.97 (0.09-0.98) |
| **Thigh** | 0.90 (0.75-0.96) | 0.81 (0.47-0.93) |
| **Leg** | 0.92 (0.80-0.97) | 0.95 (0.86-0.98) |
| **Foot** | 0.70 (0.31-0.88) | 0.90 (0.76-0.96) |

Results are Intraclass Correlation Coefficient (ICC).

Data derived from 20 SSc patients and 18 healthy controls examined, twice, two weeks apart

by TS.

**Table S2 -** Shear-wave velocity values and mRSS, per site of analysis, at baseline and follow-up, in SSc patients and controls.

|  | **SSc patients n=21** | | | **Controls n=15** | | | **SSc *vs* controls at baseline**  **(p value)^†^** | **SSc *vs* controls at follow-up**  **(p value)^†^** |
| --- | --- | --- | --- | --- | --- | --- | --- | --- |
| **Rodnan sites** | **Baseline^#^** | **Follow-up^#^** | **Baseline** ***vs* follow-up**  **(p value)^‡^** | **Baseline^#^** | **Follow-up^#^** | **Baseline *vs* follow-up**  **(p value)^‡^** |
| **Chest** | **SWV** 2.90 (2.25 - 3.25)  ***mRSS*** 0.0 (0 – 1.0) | 1.40 (1.26 – 1.52)  0.0 (0.0) | **0.000**  0.129 | 2.45 (2.35 – 2.90) | 1.46 (1.30 – 1.60) | **0.001** | NS | NS |
| **Abdomen** | **SWV** 2.20 (1.90 – 3.0)  ***mRSS*** 0.0 (0.0) | 1.41 (1.12 – 1.56)  0.0 (0.0) | **0.000**  0.083 | 1.98 (1.90 – 2.60) | 1.55 (1.30 – 1.82) | **0.001** | NS | NS |
| **Upper arm** | **SWV** 2.75 (2.42 – 3.0)  ***mRSS*** 0.0 (0.0 – 0.5) | 1.25 (1.15 – 1.39)  0.0 (0.0) | 0.000  **0.046** | 2.15 (2.0 – 2.60) | 1.29 (1.18 – 1.33) | **0.001** | **0.009** | NS |
| **Forearm** | **SWV** 2.70 (2.47 – 3.28)  ***mRSS*** 0.0 (0.0 – 1.0) | 1.47 (1.27 – 1.87)  0.0 (0.0) | **0.000**  **0.024** | 2.20 (2.03 – 2.55) | 1.37 (1.20 – 1.53) | **0.001** | **0.001** | NS |
| **Hand** | **SWV** 3.24 (2.55 – 4.32)  ***mRSS*** 1.0 (0.0 – 1.5) | 2.25 (1.95 – 2.76)  0.0 (0.0 – 1.0) | **0.000**  0.09 | 2.25 (2.0 – 2.48) | 1.69 (1.38 – 1.83) | **0.001** | **0.0001** | **0.001** |
| **Finger** | **SWV** 3.35 (2.70 – 5.12)  ***mRSS*** 2.0 (1.0 – 2.0) | 2.51 (2.17 – 7.31)  2.0 (1.0 – 2.5) | 0.881  0.276 | 2.25 (2.15 – 2.4) | 1.72 (1.44 – 1.97) | **0.001** | **0.0001** | **0.001** |
| **Thigh** | ***SWV*** 2.35 (2.22 – 2.80)  ***mRSS*** 0.0 (0.0 – 0.0) | 1.47 (1.37 – 1.70)  0.0 (0.0 – 0.0) | **0.000**  0.317 | 2.12 (2.03 – 2.26) | 1.44 (1.35 – 1.56) | **0.001** | **0.002** | NS |
| **Leg** | **SWV** 2.85 (2.28 – 3.9)  ***mRSS*** 0.0 (0.0 – 1.0) | 2.05 (1.79 – 2.44)  0.0 (0.0) | **0.003**  0.206 | 2.5 (2.23 – 2.8) | 2.24 (1.95 – 2.51) | 0.187 | NS | NS |
| **Foot** | **SWV** 2.72 (2.38 – 4.21)  ***mRSS*** 0.0 (0.0 – 1.0) | 1.67 (1.58 – 2.03)  0.0 (0.0) | **0.000**  0.132 | 2.38 (2.07 – 2.45) | 1.53 (1.27 – 1.85) | **0.001** | **0.003** | NS |

**^#^**Values are in median (IQR)**. ^‡^**Wilcoxon signed-rank test**. ^†^**Mann–Whitney U test. NS, Non-significant. Statistically significant results are in bold**.**

**Table S3 -** Shear-wave velocity values, at Rodnan skin sites, between baseline and follow-up, in the patients with SSc according to clinical skin phase of the disease.

|  |  | **Oedematous to Fibrotic /Atrophic^#^** | **Fibrotic to Fibrotic^#^** | **Fibrotic to Atrophic^#^** | **Differences between groups^†^**  **(p value)** | | |
| --- | --- | --- | --- | --- | --- | --- | --- |
|  | **Age, years^#^** | 55.0 (41.5-61.0) | 58.0 (51.0-63.0) | 62.0 (46.0-73.0) |  |  |  |
|  | **Disease duration at baseline, years^#^** | 4.0 (1.0-9.0) | 10.0 (8.0-10.0) | 15.0 (13.0-25.0) | **A *vs* B** | **A *vs* C** | **B *vs* C** |
| **Chest** | Baseline | 3.0 (2.4-4.4) | 2.9 (2.0-3.2) | 2.7 (2.3-3.4) | NS | NS | NS |
|  | Follow-up | 1.5 (1.2-1.6) | 1.4 (1.3-1.5) | 1.4 (1.1 – 1.7) | NS | NS | NS |
|  | **Change** | -51.9% (-62.3 to -45.6) | -51.5% (-54.8 to -36.3) | -46.5% (-57.7 to -41.2) | NS | NS | NS |
| **Abdomen** | Baseline | 3.3 (2.7-3.6) | 2.0 (1.8-2.4) | 2.2 (2.0-3.0) | **0.003** | NS | NS |
|  | Follow-up | 1.4 (1.0-1.7) | 1.2 (1.1-1.5) | 1.5 (1.3 – 1.7) | **0.006** | NS | NS |
|  | **Change** | -57.3% (-60.8 to -52.8) | -32.2% (-49.1 to -5.2) | -30.9% (-48.1 to -23.9) | **0.05** | NS | NS |
| **Upper arm** | Baseline | 3.6 (2.9-5.5) | 2.5 (2.0-2.8) | 2.6 (2.5-2.9) | **0.003** | **0.016** | NS |
|  | Follow-up | 1.2 (1.1-1.5) | 1.4 (1.3-1.9) | 1.2 (1.0 – 1.3) | NS | NS | NS |
|  | ***Change*** | -61.5% (-74.8 to -57.7) | -43.0% (-53.9 to -34.4) | -53.2% (-61.5 to -50.8) | **0.001** | NS | NS |
| **Forearm** | Baseline | 3.3 (3.1-5.9) | 2.6 (2.2-3.0) | 2.5 (2.4-2.7) | **0.013** | **0.008** | NS |
|  | Follow-up | 2.1 (1.5-3.7) | 1.5 (1.3-1.9) | 1.4 (1.0 – 1.5) | **0.018** | NS | NS |
|  | ***Change*** | -46.6% (-64.7 to -30.2) | -41.9% (-55.2 to -31.3) | -51.9% (-57.5 to -36.6) | NS | NS | NS |
| **Hand** | Baseline | 4.6 (3.4-5.5) | 2.8 (2.4-3.3) | 3.7 (2.4- 5.0) | **0.038** | NS | NS |
|  | Follow-up | 2.77 (2.3-3.0) | 1.9 (1.6-2.3) | 2.2 (1.9 – 2.9) | **0.03** | NS | NS |
|  | ***Change*** | -32.3% (-62.9 to -21.4) | -32.3% (-47.7 to -19.2) | -44.7% (-52.3 to 3.6) | NS | NS | NS |
| **Finger** | Baseline | 4.8 (3.4-5.5) | 2.9 (2.4-4.7) | 3.0 (2.7 – 5.5) | NS | NS | NS |
|  | Follow-up | 7.68 (3.8-5.6) | 2.3 (2.0-3.4) | 2.3 (1.9 – 5.8) | **0.04** | NS | NS |
|  | ***Change*** | 59.7% (4.5 to 87.1) | -13.7% (-46.2 to 8.2) | -15.5% (-37.6 to 21.4) | NS | NS | NS |
| **Thigh** | ***Baseline*** | 2.5 (2.3-3.2) | 2.4 (2.2-2.8) | 2.4 (2.1 – 2.4) | NS | NS | NS |
|  | Follow-up | 1.6 (1.4-1.7) | 1.4 (1.3-1.6) | 1.45 (1.4-1.6) | NS | NS | NS |
|  | ***Change*** | -40.9% (-48.0 to -31.0) | -38.1% (-46.3 to -30.0) | -34.1% (-39.7 to -29.5) | NS | NS | NS |
| **Leg** | Baseline | 4.0 (3.1-4.8) | 2.7 (2.3-3.5) | 2.3 (2.0 – 3.1) | NS | **0.032** | NS |
|  | Follow-up | 1.8 (1.7-3.7) | 2.1 (1.8-2.4) | 2.0 (1.9-2.5) | NS | NS | NS |
|  | ***Change*** | -28.4% (-61.3 to -14.0) | -30.5% (-46.5 to -4.7) | -24.9% (-31.8 to 21.5) | NS | NS | NS |
| **Foot** | Baseline | 4.4 (3.2-4.9) | 2.5 (2.3-3.5) | 2.6 (2.3 – 4.2) | **0.019** | NS | NS |
|  | Follow-up | 1.7 (1.5-2.0) | 1.6 (1.5-1.9) | 1.9 (1.6-1.9) | NS | NS | NS |
|  | ***Change*** | -61.1% (-65.1 to -23.8) | -36.4% (-49.0 to -33.7) | -29.7% (-53.1 to -21.7) | NS | NS | NS |

^#^Values are in median (Q1-Q3).

^‡^Wilcoxon signed-rank test.

^†^Mann–Whitney U test.

**NS,** Non-significant. Statistically significant results are in bold.

**A:** Oedematous to Fibrotic (n=3)/Atrophic (n=2);

**B:** Fibrotic to Fibrotic (n=11)

**C:** Fibrotic to Atrophic (n=5)

**Table S4** – Shear-wave velocity values at Rodnan sites with clinically unaffected skin (i.e. mRSS=0) and in controls, at baseline and follow-up.

|  | **SSc patients**  **(n=21)** | | | **Controls**  **(n=15)** | | | **SSc *vs* controls at baseline**  **(p value)^†^** | **SSc *vs* controls at follow-up**  **(p value)^†^** |
| --- | --- | --- | --- | --- | --- | --- | --- | --- |
| **Rodnan sites** | **Baseline^#^** | **Follow-up^#^** | **Baseline *vs* follow-up**  **(p value)^‡^** | **Baseline^#^** | **Follow-up^#^** | **Baseline *vs* follow-up**  **(p value)^‡^** |
| **Chest** | 2.7 (2.0 – 3.2)  ***n=15*** | 1.3 (1.2 – 1.5) | **0.001** | 2.45 (2.35 – 2.90) | 1.46 (1.30 – 1.60) | **0.001** | NS | NS |
| **Abdomen** | 2.2 (1.87 – 2.9)  ***n=18*** | 1.32 (1.11 – 1.54) | **0.0001** | 1.98 (1.90 – 2.60) | 1.55 (1.30 – 1.82) | **0.001** | NS | NS |
| **Upper arm** | 2.52 (2.13 – 2.83)  ***n=16*** | 1.24 (1.14 – 1.39) | **0.0001** | 2.15 (2.0 – 2.60) | 1.29 (1.18 – 1.33) | **0.001** | NS | NS |
| **Forearm** | 2.57 (2.41 – 2.7)  ***n=10*** | 1.44 (1.21 – 1.7) | **0.005** | 2.20 (2.03 – 2.55) | 1.37 (1.20 – 1.53) | **0.001** | **0.055** | NS |
| **Hand** | 2.45 (2.3 – 3.0)  ***n=7*** | 1.9 (1.5 – 2.3) | **0.068** | 2.25 (2.0 – 2.48) | 1.69 (1.38 – 1.83) | **0.001** | NS | NS |
| **Finger** | 2.5 (2.3 – 2.5)  ***n=2*** | 1.84 (1.5 – 1.84) | - | 2.25 (2.15 – 2.4) | 1.72 (1.44 – 1.97) | **0.001** | NS | NS |
| **Thigh** | 2.35 (2.2 – 2.5)  ***n=18*** | 1.4 (1.36 – 1.55) | **0.0001** | 2.12 (2.03 – 2.26) | 1.44 (1.35 – 1.56) | **0.001** | **0.007** | NS |
| **Leg** | 2.53 (2.27 – 3.55)  ***n=15*** | 2.0 (1.8 – 2.8) | **0.002** | 2.5 (2.23 – 2.8) | 2.24 (1.95 – 2.51) | 0.187 | NS | NS |
| **Foot** | 2.45 (2.36 – 3.05)  ***n=13*** | 1.6 (1.58 – 1.91) | **0.005** | 2.38 (2.07 – 2.45) | 1.53 (1.27 – 1.85) | **0.001** | **0.022** | NS |

**^#^**Values are in median (Q1-Q3)

**^‡^**Wilcoxon signed-rank test

**^†^**Mann–Whitney U test. NS: Non-significant. Statistically significant results are in bold.

**Table S5** – Percentage changes in shear-wave velocity values at Rodnan sites with unaffected skin at baseline (i.e. mRSS=0) and in controls, between baseline and end of follow-up.

| **Rodnan sites** | **SSc patients**  **(n=21)^#^** | **Controls**  **(n=15)^#^** | **SSc *vs* controls**  **(p value)^†^** |
| --- | --- | --- | --- |
| **Chest** | -50.0% (-53.6 to -41.7) | -46.9% (-50.0 to -32.0) | NS |
| **Abdomen** | -35.8% (-57.7 to -28.7) | -32.6% (-43.9 to -8.2) | NS |
| **Upper arm** | **-52.5% (-58.8 to -40.7)** | **-41.5% (-48.7 to -38.8)** | **0.03** |
| **Forearm** | -46.2% (-57.5 to -40.7) | -37.1% (-52.0 to -23.3) | NS |
| **Hand** | -21.8% (-52.6 to -19.9) | -26.2% (-38.6 to -8.5) | NS |
| **Finger** | -5.6% (-44.5 to -5.6) | -24.6% (-29.8 to -12.4) | NS |
| **Thigh** | -36.1% (-45.3 to -30.3) | -31.6% (-37.3 to -24.0) | NS |
| **Leg** | **-28.4% (-37.8 to -3.2)** | **-10.3% (-27.3 to -6.9)** | **0.05** |
| **Foot** | -35.0% (-51.7 to -22.3) | -27.1% (-45.2 to -14.3) | NS |

^#^Values are in median (Q1-Q3).

^†^Mann–Whitney U test. NS, Non-significant. Statistically significant results are in bold.

**Table S6** - Shear-wave velocity values, at Rodnan skin sites, at baseline and follow-up, in patients with limited vs diffuse SSc.

|  | **Limited (N=12)** | | | **Diffuse (N=9)** | | |  |  |  |
| --- | --- | --- | --- | --- | --- | --- | --- | --- | --- |
|  | **Baseline** | **Follow-up** | **%Change** | **Baseline** | **Follow-up** | **%Change** | **Baseline Lim vs Dif**  **(p value^†^)** | **Follow-up Lim vs Dif**  **(p value^†^)** | **% Change Lim vs Dif**  **(p value^†^)** |
| **Chest** | 2.9 (2.4-3.3) | 1.3 (1.2-1.5) | -52.3 (-56.0 - -45.4) | 2.4 (2.1-3.4) | 1.4 (1.4-1.6) | -41.3 (-54.5 - -33.6) | 0.602 | 0.09 | 0.118 |
| **Abdomen** | 2.1 (1.8-2.8) | 1.2 (1.1-1.5) | -35.8 (-58.5 - -29.0) | 2.5 (1.9-3.4) | 1.5 (1.3-1.7) | -48.9 (-58.1 - -24.1) | 0.31 | 0.247 | 0.943 |
| **Upper arm** | 2.5 (2.0-2.9) | 1.2 (1.1-1.3) | -53.5 (-58.9 - -44.7) | 2.9 (2.6-3.6) | 1.4 (1.2-1.6) | -51.6 (-64.4 - -36.4) | **0.05** | **0.02** | 0.943 |
| **Forearm** | 2.6 (2.0-2.9) | 1.4 (1.2-1.7) | -46.6 (-57.3 - -42.5) | 3.3 (2.5-4.0) | 1.9 (1.4-2.2) | -40.8 (-64.7 - -28.7) | 0.09 | **0.03** | 0.470 |
| **Hand** | 3.0 (2.4-3.6) | 2.2 (1.6-2.5) | -34.5 (-45.7 - -9.0) | 4.6 (2.9-6.3) | 2.8 (2.0-3.0) | -32.3 (-57.3 - -24.8) | **0.04** | **0.03** | 0.425 |
| **Finger** | 2.9 (2.7-3.3) | 2.3 (2.0-2.6) | -21.8 (-46.0 - 6.3) | 5.4 (3.5-6.8) | 7.6 (2.8-8.7) | 14.2 (-22.3 - 70.9) | **0.006** | **0.007** | 0.074 |
| **Thigh** | 2.3 (2.3-2.5) | 1.4 (1.4-1.5) | -43.2 (-46.0 - -32.7) | 2.4 (2.2-3.2) | 1.6 (1.5-1.8) | -34.9 (-39.5 - -28.9) | 0.808 | **0.03** | 0.305 |
| **Leg** | 2.6 (3.1-4.8) | 2.0 (1.9-2.2) | -25.3 (-32.9 - -6.4) | 3.1 (2.5-4.8) | 2.3 (1.8-3.3) | -28.4 (-60.0 - 12.5) | 0.169 | 0.72 | 0.744 |
| **Foot** | 2.7 (2.3-3.9) | 1.6 (1.5-1.9) | -43.4 (-54.9 - -31.1) | 2.9 (2.4-4.7) | 1.9 (1.6-2.1) | -33.8 (-60.0 - -20.9) | 0.521 | 0.27 | 0.558 |

^#^Values are in median (Q1-Q3).

^†^Mann–Whitney U test. NS, Non-significant. Statistically significant results are in bold.

Limited form: median 60.5 years old; median disease duration of 10.0 (0-30) years.

Diffuse form: median 51.0 years old; median disease duration of 9.0 (1-13) years.


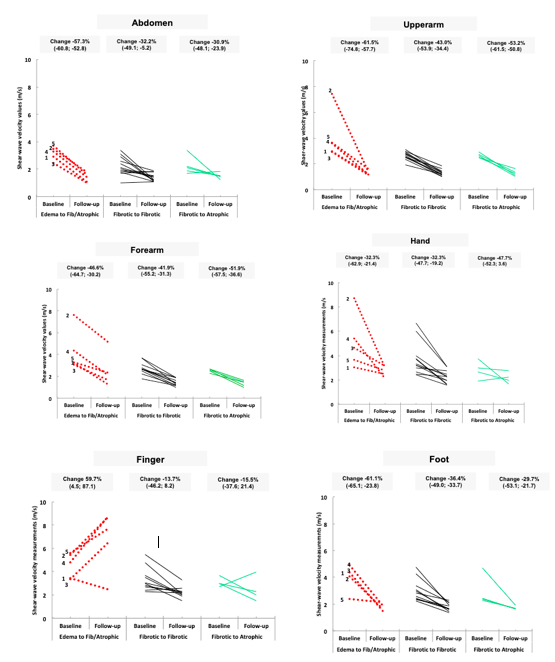
**Figure S1 –** Shear-wave velocity values in the skin Rodnan sites at baseline and follow-up, in SSc patients, according the progression of the skin phase of the disease. Patients in an oedematous phase progressed to a fibrotic (n=3) or atrophic phase (n=2); 11 maintained the fibrotic phase; and 5 progressed from a fibrotic to an atrophic phase. Percentage change values are in medians (Q1-Q3).
